# Exploring the mechanisms of collaboration between the Tuberculosis and Diabetes Programs for the control of TB-DM Comorbidity in Ghana

**DOI:** 10.1186/s13104-021-05637-1

**Published:** 2021-05-31

**Authors:** Rita Suhuyini Salifu, Khumbulani W. Hlongwana

**Affiliations:** 1 Discipline of Public Health Medicine, School of Nursing and Public Health, University of KwaZulu-Natal, Durban, South Africa; 2 Health and Development Solutions Network, Tamale, Ghana

**Keywords:** Ghana, Policy, Qualitative research, National TB control program, NCD control program

## Abstract

**Objectives:**

To explore the mechanisms of collaboration between the stakeholders, including National Tuberculosis Control Program (NTP) and the Non-Communicable Disease Control and Prevention Program (NCDCP) at the national, regional, and local (health facility) levels of the health care system in Ghana. This is one of the objectives in a study on the “Barriers and Facilitators to the Implementation of the Collaborative Framework for the Care and Control of Tuberculosis and Diabetes in Ghana”

**Results:**

The data analysis revealed 4 key themes. These were (1) Increased support for communicable diseases (CDs) compared to stagnant support for non-communicable diseases (NCDs), (2) Donor support, (3) Poor collaboration between NTP and NCDCP, and (4) Low Tuberculosis-Diabetes Mellitus (TB-DM) case detection.

**Supplementary Information:**

The online version contains supplementary material available at 10.1186/s13104-021-05637-1.

## Introduction

Non-communicable diseases (NCDs) have been described as a “chronic emergency” requiring urgent global public health action, particularly in low- and middle -income countries (LMICs) [1]. In Africa alone, projections suggest that 23.9 million individuals will be living with diabetes mellitus (DM) by the year 2030 [1]. Given the existing high tuberculosis (TB) burden in LMICs, a simultaneous parallel increase in DM prevalence is expected [2]. This growing public health problem has caught the attention of the global community, culminating in the development of 2030 global strategy aimed at reducing TB incidence and TB mortality by 80% and 90%, respectively [3] Despite these initiatives, the African continent has the second-highest TB burden in the world [3] and Ghana has not been spared from the ravages of the TB burden [3]. In Ghana, the national TB prevalence is 148/100,000 population [3], a situation that is exacerbated by the growing TB-DM co-epidemic, thereby imposing a strain on the health care systems. This phenomenon calls for a radical review of the vertical nature of TB and DM programs in the country [4].

In 2011, the World Health Organization (WHO) and the International Union Against Tuberculosis and Lung Disease (Union) took up the challenge of the rising TB-DM co-epidemic inherently linked to the vertical nature of the TB and DM programs by launching the collaborative framework for care and control of TB and DM [5].

The initiative by the WHO and Union was not new in the Ghanaian context since a comparable country-specific programme had been established seventeen years (1994) earlier. Ghana established the National Tuberculosis Control Program (NTP), as part of the disease control department under the public health division of the Ghana Health Service (GHS), with funding from the government and donor funds [6] and that was some years before the launch of the WHO’s collaborative framework, which took place in 2011. Needless to say, the continued pre-2011 increase in TB-DM comorbidity in Ghana raised questions about the effectiveness of the 1994 program. To date, the NTP in Ghana is responsible for the policy interventions, such as the WHO-Union collaborative framework and proposes policies that the Ministry of Health (MoH) can implement to control TB [7].

Over the past decade, NCDs have become a priority in global health agenda, and Ghana, which has been experiencing a steady increase in NCDs, is no exception [1]. The formation of the NCD Control and Prevention Program (NCDCP) in 1992, under the wing of the GHS, is a sign that Ghana has grappled with these public health challenges for a long time [8]. The program’s mandate is to plan, develop clinical practice guidelines, train, advocate and coordinate NCD activities [8]. Ghana's priority NCDs include hypertension, diabetes, cancers and the chronic respiratory diseases, genetic disorders (sickle cell disease) and injuries [8].

Despite notably limited individual TB/ DM program advancement, the vertical nature of these programs limits the potential contribution of implementing collaborative plans, particularly using the tools provided by the WHO-Union collaborative framework.

This study sought to explore the mechanisms of collaboration between the stakeholders, including NTP and the NCDCP at the national, regional, and local (health facility) levels of the health care system in Ghana.

## Main text

### Methods

Study Setting—This study took place in both Accra, the country’s national capital and the Northern Region (NR) of Ghana. Accra is the capital city of Ghana, where national offices of the NTP and the NCDCP are located. The Northern Regional offices of these institutions are in Tamale, the regional capital and the TB and DM units are located at the three health facilities in the Northern region.

Study Design- Guided by the grounded theory design, this study explored the mechanisms of collaboration between NTP and NCDCP programs in Ghana at the levels of national, regional and health facilities, using key- informant interviews. Ethical approval was obtained from both the University of KwaZulu-Natal (UKZN) Biomedical Research Ethics Committee (BREC) (BE262/19) and Ghana Health Service Ethics Review Committee (GHSERC) (GHS-ERC 012/04/19). The researcher obtained permission and introductory letters from the GHS and presented at the NTP and NCDCP national and regional offices in Accra and Tamale, respectively, as well as the three health facilities in the Northern Region. As a gesture of study endorsement, the directors of the programs introduced the researcher to the program officers who met the selection criteria for inclusion into the study. At the health facilities, the medical directors introduced the researcher to the staff of TB and DM units.

Data generation—Twelve (12) purposively selected key informants were interviewed by the researcher, face-to-face at two locations, namely: Accra and Tamale. Two participants were recruited from NTP (national and regional), two from the NCDCP (national and regional) and eight participants from the three health facilities. All respondents read the information sheet and signed informed consent prior to being interviewed. In-depth interviews lasted for 30–45 min and were audio-recorded (with participants’ permission). Two participants consented to the study, but objected to being audio-recorded, hence their interviews were only recorded through the hand-written notes. All the interviews were conducted in English, as all the participants were comfortable with English language. The researcher also took the summary notes during all the interviews. The semi-structured interview guides (Additional files 1 and 2) covered questions on existing policies to establish the mechanism of collaboration, state of TB-DM at both the national and regional levels, stakeholder inputs into the policy process and coordination of activities between NTP and NCDCP.

Data Analysis—Using the grounded theory analytical approach, the digital audio recordings from the interviews were carefully transcribed in verbatim by a professional transcriber and the researcher, who has training in qualitative research methods, verified transcriptions, to confirm data quality. Furthermore, the researcher meticulously read through all the transcripts and compared them with field notes and audio-records, to ensure the integrity of data. Recurring ideas were grouped into themes, and emerging themes were coded manually by the researcher. Similar codes were grouped under major themes—the analysis generated 4 main themes.

### Results

The key informants in this study were staff of the NTP and NCDCP at the national and regional offices, Hospital managers and TB and DM Unit managers in three health facilities. A total of 12 participants comprised of National program managers (n = 2), Regional program managers (n = 2), Hospital managers (n = 2), TB unit manager (n = 3), and DM unit managers (n = 3) (Table 1).

**Table 1:** Characteristics of the study participants

	Participant	Age range	Years of experience	Stakeholder
Policy makers	Participant 1	35–44	9	NTP
	Participant 2	35–44	10	NCDCP
	Participant 3	45–54	25	NTP
	Participant 4	45–54	15	NCDCP
Implementers	Participant 5	25–34	9	Hospital TB Unit
	Participant 6	35–44	9	Hospital NCD Unit
	Participant 7	35–44	12	Hospital NCD Unit
	Participant 8	45–54	23	Hospital manager
	Participant 9	55–64	26	Hospital Manager
	Participant 10	55–64	33	Hospital TB Unit
	Participant 11	55–64	35	Hospital TB Unit
	Participant 12	55–64	35	Hospital NCD Unit

Participants’ responses reflected their perception of the extent of integration, challenges, and opportunities to implementing the collaborative framework (Table 2). The main themes from the analysis were (1) Increased support for communicable diseases compared to stagnant support for non-communicable diseases, (2) Donor support (3), Poor collaboration between NTP and NCDCP, and (4) Low TB-DM Case detection.

**Table 2:** Themes and quotes from participants on mechanism of collaboration

Themes	Comments/quotes
Increased support for communicable diseases compared to stagnant support for non-communicable diseases	"In some facilities, the NTP brought in task shifting officers because screening is a role the nurses are supposed to do, but they are saying it is another load, so we are shifting it. The aim is that they see it as their routine work". (Participant 1)
	“The other policy is to have separate clinics for DM apart from the normal OPD [outpatient department], but because of human resource challenges, not all health facilities are able to do this”. (Participant 4)
	The standard thing is to screen patients for DM, but it is expensive, so no hospital can do that because of the strips. It should have been part of the insurance package, especially if you are coming for the first time you are checked for DM. We should deliberately make it a policy that DM is checked, just as blood pressure”. (Participant 4)
	“One of the initiatives is the wellness clinic [for NCDs]. We asked all hospitals to set up the wellness clinic. The challenge is how to fund it.” (Participant 4)
	“The whole NCDCP itself is not even funded so what are you going to use to follow-up [TB-DM cases]?” (Participant 2)
Donor support	“Most support from partners go to maternal and child health, not NCD'S. Depending on what the partners prioritize, that is where the money goes ". (Participant 4)
	“We [NCDCP] rely on partners because we don't have any direct source of funding for the program, these collaborations become essential. If you intend to get anything done, you need funding." (Participant 2)
	Another challenge is funding. We get support from the WHO and the government of Ghana, but the main support is from the Global Fund.” (Participant 3)
Poor collaboration between TB and DM programs	“There is currently no collaboration between the NCDCP and TB programs. There are ongoing talks, now issues have come up because of the Malaria-HIV-TB funding structure. They are directly global funded, and the way their lines of funding are, most times, they are stuck on those activities and don't veer horizontally to other programs." (Participant 2)
	“No, we do not have any collaboration with the TB unit, we have not had any meetings in that direction. I do not remember being invited to any TB workshop”. (Participant 7)
	"We are not seeing collaboration from the diabetic side. We [NTP] say they should screen, but they should also know it is for the best management ". (Participant 1)
	“It will be much smoother from the TB side to get things along than the DM side. A lot of hospitals have done that in-house usually based on personal initiatives. It’s not a nationwide thing”. (Participant 2)
Low TB-DM case detection	“We [NTP] have not really zoned into TB-DM screening, our challenge is case detection, but from where I am sitting, the screening among the DM [patients] is very poor and because most of the places do not have a specialized diabetic clinic, it's difficult to detect. Since they all pass through the OPD”. (Participant 3)
	“In the few places where there are diabetic clinics, it’s easier to implement but, in most places, where they go through the OPD its likely to miss them [TB-DM] because not everyone is asked questions related to DM”. (Participant 2)
	“We are interested in our TB cases, so we are asking the DM clinic to screen but the other way round we are not screening for DM, so we have no data. We always talk of looking for TB comorbidity but testing for DM is not captured in our register”. (Participant 1)
	“We need to show more evidence of the TB-DM data and provide feedback to practitioners”. (Participant 3)
	We know risk factors for TB is HIV and DM, so we have included screening at DM clinics.” (Participant 1)
	“Because it is difficult to segregate the data it’s difficult to get TB-DM cases” (Participant3)
	“There is no such tool for screening TB patients for DM (participant 4)
	The success of policies is on engaging the people and making sure they have the capacity to do it, the large numbers and workload give the health workers stress”. (Participant 2)
	The emphasis should be more on screening the TB cases for DM rather than DM for TB. When it's the other way around, we need more orientation. Most of the comorbidity cases have been picked up from the TB clinic. TB clinics are fairly well run in Ghana because of the HIV-TB structures. (Participant 2)

The results of this study revealed that NTP introduced task-shifting officers in some facilities to circumvent the additional burden imposed by screening on the nurses (Participant 1).

Research participants who were the custodians of the policy (national level) identified the dependency on donor support to fund the NCDCP as one of the key challenges, which negatively affected the TB-DM collaboration (Participant 2). The fact that donor support was mainly targeted at health conditions, like maternal and child health, did not advance the agenda of TB-DM collaboration (Participant 4).

Findings from this study showed no evidence of collaboration between the NTP and NCDCP at the national and regional levels (Participant 2, & 7). However, at the facility level, some pockets of informal collaboration were observed, however, these were based on the healthcare workers’ personal initiatives (Participant 2).

Respondents attributed low TB-DM detection to poor screening among DM patients (Participants 2), limited DM clinics in most health facilities (participant 1) and limited data for TB-DM comorbidity, mainly due to the lack of designated diabetic clinics in most health facilities (participant 3).

### Discussion

To the best of our knowledge, this is the first study to explore the mechanisms of collaboration between the NTP and NCDCP at the national, regional, and local levels, using qualitative methods.

Health care systems in resource-constrained settings are grappling with responding to the co-existence of both CDs and NCDs in the same populations or individuals, hence our findings have reiterated that there still exists an imbalance between health delivery for CDs and NCDs in Ghana [9]. The findings of this study found that the delivery of care was biased towards CDs. This was supported by views expressed in this study showing that NCDs were not a top priority as CDs. These findings are consistent with the results of a study previously conducted in Ghana, confirming that diagnostic and care services to infectious diseases are more accessible than that of NCDs[10]. This may be due to the health system structure in Ghana, which is generally skewed towards managing CDs, a phenomenon that is not peculiar to the Ghanaian context, as it has also been reported in other low-to-middle-income countries [10–12]. In Ethiopia and India, there was poor quality of care for DM patients, and NCD services lacked systems for monitoring and evaluation [13, 14]. Along the same lines, Critchley and colleagues [15] shared that there is considerable support and funding for TB treatment by the WHO, and this is in contrast to financially deprived DM care in LMICs [15]. Limited health insurance is available for DM care in LMICs, leading to high out of pocket cost and poor outcomes in detection and management of DM patients [15, 16]. Further studies in Ghana and Bangladesh highlighted the lack of infrastructure, guidelines, and supplies to be able to deliver NCD care [10, 12].This study found that healthcare workers were concerned that screening of patients was an additional responsibility to their existing workload, and this was consistent with the findings of other studies [16]. However, in Ghana, the NTP with funding from the Global Fund augmented this by recruiting extra staff as task shifting officers to support the integration.

According to participants’ views, donor support was a critical source of funding to complement the limited budget of the MoH/GHS. This funding from donors was largely tied to specific health conditions and outcomes, which influenced the strategic direction of the diseases program in receipt of the funding. The reliance on donor support may be due to the limited national budgets for health ministries which were not a priority for funding in comparison with other ministries as observed by Puchner et al. [2]. In Kyrgyzstan, funding for TB was 44% financed by external donors with diabetes receiving no direct external funding [17].

The low TB-DM case detection may be due to the limited collaboration between the NTP and NCDCP at all levels of the healthcare system. This research found that integration at the program levels was largely hindered because of sources of funding and structured objectives of the programs, which did not leave room for integrating other health conditions. Therefore, funding appears to be one of the most important factors that would influence the mechanism of collaboration between NTP and NCDCP. Currently, the screening for DM is seen as burdensome due to poor funding of DM programs.

Additionally, the low awareness of the TB-DM comorbidity and how to integrate services was adversely affecting the rate of case detection, as reported in other research conducted in Ghana [18]. According to a publication on Ghana's NCD policy, poor screening is also attributable to low awareness of implementers in the northern region [19].

#### Conclusions

There exist limited mechanisms of collaborations between the NTP and NCDCP mainly at the health facility level, with the lack of funding identified as one of the important impediments. There is still a gap at the national and regional program levels with regards to joint coordination of activities geared towards TB and DM collaboration. Therefore, the results of this study can be used to advocate for better TB-DM collaboration and lobby policymakers and politicians to prioritise integrative services for these two health conditions. Development of different health insurance schemes for DM patients may potentially help support in the high cost of DM care, as suggested by some study participants. Further awareness and training are required on the TB-DM comorbidity, and the recommendations by the WHO collaborative framework for the care and control of TB and DM will promote further integration. Lastly, there is a need for advocacy groups to highlight the importance of prioritizing DM, alongside TB, in donor funding, to circumvent low uptake of DM screening services and improve DM health outcomes.

## Limitations

- This study was confined to key informants and may have benefited from a larger group of stakeholders working in the non-governmental health sector.
- The study was unable to explore the collaboration of the TB and DM programs at the district and sub district levels of Ghana.

## Supplementary Information

**Additional file 1.** Interview guide 1-Health Managers.

**Additional file 2.** Interview guide 2- Policy Makers.

## Data Availability

All data generated or analyzed during this study are included in this published article [and its additional files].

## Abbreviations

CDs: Communicable Diseases
DM: Diabetes mellitus
GHS: Ghana Health Service
HIV: Human Immunodeficiency Virus
LMICs: Low- and Middle-Income Countries
MoH: Ministry of Health
NCDs: Non-Communicable Diseases
NCDCP: Non-communicable Disease Control and Prevention Program
NTP: Ghana National Tuberculosis Control Program
TB: Tuberculosis
TB-DM: Tuberculosis-Diabetes
Union: International Union Against Tuberculosis and Lung Disease
WHO: World Health Organization

## References

[1] Bosu WK (2013). Accelerating the control and prevention of non-communicable diseases in Ghana: the key issues. Postgraduate Med J Ghana.

[2] Puchner KP, Rodriguez-Fernandez R, Oliver M, Solomos Z (2019). Non-communicable diseases and tuberculosis: anticipating the impending global storm. Glob Public Health.

[3] WHO (2019). Global tuberculosis report World Health Organization.

[4] Harries A, Kumar A, Satyanarayana S, Lin Y, Zachariah R, Lönnroth K (2015). Diabetes mellitus and tuberculosis: programmatic management issues. Int J Tuberc Lung Dis.

[5] WHO (2011). Collaborative framework for care and control of tuberculosis and diabetes.

[6] Amo-Adjei J (2014). Political commitment to tuberculosis control in Ghana. Glob Public Health.

[7] Amo-Adjei J, Awusabo-Asare K (2013). Reflections on tuberculosis diagnosis and treatment outcomes in Ghana. Arch Public Health.

[8] Bosu WK (2012). A comprehensive review of the policy and programmatic response to chronic non-communicable disease in Ghana. Ghana Med J.

[9] Marais BJ, Lönnroth K, Lawn SD, Migliori GB, Mwaba P, Glaziou P (2013). Tuberculosis comorbidity with communicable and non-communicable diseases: integrating health services and control efforts. Lancet Infect Dis.

[10] Kushitor MK, Boatemaa S (2018). The double burden of disease and the challenge of health access: evidence from access, bottlenecks, cost and equity facility survey in Ghana. PLoS ONE.

[11] Temu F, Leonhardt M, Carter J, Thiam S (2014). Integration of non-communicable diseases in health care: tackling the double burden of disease in African settings. Pan Afr Med J.

[12] Rawal LB, Kanda K, Biswas T, Tanim MI, Poudel P, Renzaho AM (2019). Non-communicable disease (NCD) corners in public sector health facilities in Bangladesh: a qualitative study assessing challenges and opportunities for improving NCD services at the primary healthcare level. BMJ Open.

[13] Sharma P, Visnegarwala F, Tripathi V (2014). Burgeoning double burden of tuberculosis and diabetes in India: magnitude of the problem–Strategies and solutions. Clin Epidemiol Global Health.

[14] Workneh MH, Bjune GA, Yimer SA (2016). Assessment of health system challenges and opportunities for possible integration of diabetes mellitus and tuberculosis services in South-Eastern Amhara Region, Ethiopia: a qualitative study. BMC Health Serv Res.

[15] Critchley JA, Restrepo BI, Ronacher K, Kapur A, Bremer AA, Schlesinger LS (2017). Defining a research agenda to address the converging epidemics of tuberculosis and diabetes: part 1: epidemiology and clinical management. Chest.

[16] Riza AL, Pearson F, Ugarte-Gil C, Alisjahbana B, van de Vijver S, Panduru NM (2014). Clinical management of concurrent diabetes and tuberculosis and the implications for patient services. Lancet Diabetes Endocrinol.

[17] Skordis-Worrall J, Round J, Arnold M, Abdraimova A, Akkazieva B, Beran D (2017). Addressing the double-burden of diabetes and tuberculosis: lessons from Kyrgyzstan. Glob Health.

[18] Obirikorang Y, Obirikorang C, Anto EO, Acheampong E, Batu EN, Stella AD (2016). Knowledge of complications of diabetes mellitus among patients visiting the diabetes clinic at Sampa Government Hospital, Ghana: a descriptive study. BMC Public Health.

[19] Nyaaba GN, Stronks K, Masana L, Larrea-Killinger C, Agyemang C (2020). Implementing a national non-communicable disease policy in sub-Saharan Africa: experiences of key stakeholders in Ghana. Health Policy Open.

