# Supplementary material for: Exploring the mechanisms of collaboration between the Tuberculosis and Diabetes Programs for the control of TB-DM Comorbidity in Ghana
Source: BMC Res Notes. 2021 May 31;14:217. doi: 10.1186/s13104-021-05637-1 (PMC8166070; doi:10.1186/s13104-021-05637-1)
Supplement: Supplementary file 1 — Additional file 1. Interview guide 1-Health Managers. [file 13104_2021_5637_MOESM1_ESM.pdf]

C. INTERVIEW GUIDE – HEALTH MANAGERS
West District, Central Regional and Tamale Teaching Hospitals

Good ……………………. My name is Rita Suhuyini Salifu, a student from School of Nursing and Public Health , University of KwaZulu-Natal. I am currently carrying out a research on the *Barriers and Facilitators to the Implementation of the Collaborative Framework for Care and Control of Tuberculosis (TB) and Diabetes (DM) in Ghana*. You have been invited as a participant in this research and I will like to have a discussion with you concerning this topic. I will need between 45-60minutes of your time. You are free to opt of the interview now or at any point in time you don't feel comfortable or skip questions you don't feel comfortable answering. You can also be rest assured that your identity or name as a respondent will not appear anywhere in the study, it will be kept confidential. I will however take down notes and a digital voice recorder to be able to cross check later with the notes to be sure we captured the right information. However, you are free to reject to any of them or both. Thank you.

Introduction

- Kindly tell me about your current role in this health facility? For example, describe your typical day at work, what do you do?

  Probe: *How long have you worked with the Ghana Health Service and in what capacities?*

- Please share briefly about the services offered by this health facility/ department (the cases you normally see etc) .

  Probe: *Please describe your catchment area as a District/Regional/Teaching hospital,*

  Probe: *Where do you get referrals from?*

- Kindly describe your supervision style

  Probe: *can you share how this style yields results?*

  Probe: *How do you introduce new guidelines/interventions to you?*

Policies and Guidelines

- Kindly share the kinds of services you usually provide for TB and DM care in this facility?

  Probe*: Can you elaborate on the guidelines followed in the care of TB and DM patients?*

  Probe: *Describe some of the challenges faced in providing these services.*

- Could you share your understanding of what the framework aims to achieve? Kindly elaborate

  Probe: *Can you describe how this is being implemented in this health facility?*

  Probe*: How has this implementation impacted on the management /administration of this department? Please elaborate*

- How has this framework been received by your staff? Please elaborate

- Can you describe any contribution of the collaborative framework in the care of DM and TB? Please elaborate.

C. INTERVIEW GUIDE – HEALTH MANAGERS

Systems and Structures for Co-Management

- How has been your experience of TB and DM links in this facility? Please elaborate.
- Kindly describe the process followed in this facility for the screening of TB patients for DM and DM patients for TB?
- Please describe the process followed in managing a TB patient if DM is detected and vice versa?
- Can you share any contribution screening has on prevention and care of TB and DM? Please elaborate?

  Probe: *Can you share some of the challenges you face in screening for these two diseases (TB & DM)?*

- Are you aware of any collaborative activities between TB and DM care organized by the Ghana Health Service? Would you share any experience of collaborative activities in this facility?
- Please share your advice on what you think will hinder the implementation of the Collaborative Framework? Kindly elaborate
- *Kindly tell me briefly about yourself*
- *Participant code*
- *Age*
- *Educational background/training*

GENERAL IMPRESSIONS

I have attempted to pose some questions in relation to your experience in the implementation of the collaborative framework. I may not have been able to capture all the issues in relation to the subject matter. Is there anything you consider important in this respect but which I did not touch on? Can you shed some light on this?

Thank you for your time. Do you have any questions for me?
