# Supplementary material for: Exploring the mechanisms of collaboration between the Tuberculosis and Diabetes Programs for the control of TB-DM Comorbidity in Ghana
Source: BMC Res Notes. 2021 May 31;14:217. doi: 10.1186/s13104-021-05637-1 (PMC8166070; doi:10.1186/s13104-021-05637-1)
Supplement: Supplementary file 2 — Additional file 2. Interview guide 2- Policy Makers. [file 13104_2021_5637_MOESM2_ESM.pdf]

A. INTERVIEW GUIDE -POLICY MAKERS
Ministry of Health (MOH/GHS)

Good ……………………. My name is Rita Quist-Therson, a student from School of Nursing and Public Health, University of KwaZulu-Natal. I am currently carrying out a research on the *Barriers and Facilitators to the Implementation of the Collaborative Framework for Care and Control of Tuberculosis (TB) and Diabetes (DM) in Ghana*. You have been invited as a participant in this research and I will like to have a discussion with you concerning this topic. I will need between 45-60minutes of your time. You are free to opt of the interview now or at any point in time you don't feel comfortable or skip questions you don't feel comfortable answering. You can also be rest assured that your identity or name as a respondent will not appear anywhere in the study, it will be kept confidential. I will however take down notes and a digital voice recorder to be able to cross check later with the notes to be sure we captured the right information. However, you are free to reject to any of them or both. Thank you.

Introduction

- Kindly tell me about your current role in this institution? For example, describe your typical day at work, what do you do?

  ***Probe**: How long have you worked at the Ministry of Health (MOH)/ Ghana Health Service (GHS) and in what capacities?*

- Can you share briefly about the role/ function of your institution with regards to health care system in Ghana?

  ***Probe**: Please describe the areas this institution is responsible for? Kindly elaborate*

Tuberculosis and Diabetes Burden in Ghana

- Kindly share from your experience on the TB and DM overview in Ghana? And Northern Region specifically?

  ***Probe**: How would you describe the TB/DM comorbidity in Ghana? kindly elaborate*

- Please elaborate on the other organizations you collaborate with (stakeholders), when it comes to TB and DM policy and its implementation. Please elaborate .

Understanding the existing policy landscape on TB & DM in Ghana

- Can you share an overview of policy formulation process at the MOH/GHS? please elaborate

  ***Probe**: Also policy implementation process explain the policy process*

- Kindly list the priority areas of policy focus by MOH/GHS over the past five years? Please elaborate .

  ***Probe**: Can you share what is planned for the next 5 years*

- Can you list all the existing TB & DM policies in Ghana and kindly elaborate on each of these policies? (request for copies)

- Please list the various stakeholders involved in TB and DM policy development.

***Probe**: Kindly describe the roles of various stakeholders involved in TB and DM policy development. Please elaborate*

- How has the international context if any, influenced TB and DM issues/policies in Ghana? Please elaborate

WHO Collaborative Framework Implementation in Ghana

- Can you share your understanding of what the collaborative framework aims to achieve? Please elaborate

***Probe**: How are the key actors involved in and affected by the policy? Please elaborate*

- Please describe the public/stakeholder input in the policy process? Can you elaborate

***Probe**: How is this policy process facilitated?*

- Kindly elaborate on MOH/GHS role in the implementation of the collaborative framework?

***Probe**: Please share your perspective on the implementation process so far.*

Dissemination/Decentralization of Framework

- How would you describe the impact of the implementation of the collaborative framework on existing policies? Please elaborate

***Probe**:Human resource capacity?*

Collaboration of TB and DM Activities

- Please share any measures MOH/GHS has in place for coordinating TB and DM activities (Ghana/Northern region)? Please elaborate

***Probe**: Kindly share any factors that facilitate / impede this collaborative activities? Please elaborate*

- Kindly tell me briefly about yourself:
  - Participant code
  - Age
  - Educational background/training

GENERAL IMPRESSIONS

I have attempted to pose some questions in relation to your experience in the implementation of the collaborative framework. I may have not been able to capture all the issues in relation to the subject matter. Is there anything you consider important in this respect but which I did not touch on? Can you shed some light on this? Thank you for your time. Do you have any questions for me?
